# Slow Codes are symptomatic of ethically and legally inappropriate CPR policies

**DOI:** 10.1111/bioe.13396

**Published:** 2025-01-31

**Authors:** Stuart McLennan, Marieke Bak, Kathrin Knochel

**Affiliations:** ^1^ Institute of History and Ethics in Medicine, Department of Preclinical Medicine, TUM School of Medicine and Health Technical University of Munich Munich Germany; ^2^ Institute for Biomedical Ethics University of Basel Basel Switzerland; ^3^ Department of Ethics, Law and Humanities, Amsterdam UMC University of Amsterdam Amsterdam The Netherlands

**Keywords:** CPR policy, resuscitation, Slow Code, translational ethics

## Abstract

Although cardiopulmonary resuscitation (CPR) was initially used very selectively at the discretion of clinicians, the use of CPR rapidly expanded to the point that it was required to be performed on all patients having in‐hospital cardiac arrests, regardless of the underlying condition. This created problems with CPR being clearly inadvisable for many patients. Do Not Resuscitate (DNR) orders emerged as a means of providing a transparent process for making decisions in advance regarding resuscitation, initially by patients and later also by clinicians. Under hospital policies in many countries, however, CPR remains the default position for all patients having cardiac arrest in the hospital if there is no DNR order in place, regardless of whether CPR is medically indicated or in the patient's best interests. “Slow Codes” are the delayed or token efforts to provide CPR when clinicians feel CPR is futile or inappropriate. After giving a historical overview of the development and the changing use of CPR, we argue that more attention needs to be given to the cause of slow codes, namely, policies requiring CPR to be performed as the default action while simultaneously lacking implementing interventions such as advance care planning as a routine policy. This is ethically and legally inappropriate, and hospital policies should be modified to allow clinicians to consider whether CPR is appropriate at the time of arrest. Such a change requires a stronger emphasis on early recognition of patients for whom CPR is not in their best interests and to improve hospital emergency planning.

While the struggle against death is an important goal of medicine, it would be a mistake to over‐emphasize this struggle; medicine is fundamentally unable to delay death forever. This is recognized in the controversial practice of initiating ‘slow codes’, which refers to the delayed or token effort to resuscitate patients when cardiopulmonary resuscitation (CPR) is thought to be futile or not in a patient's best interests. Hospital policies in many countries require the provision of CPR as the default action for patients having cardiac arrest.^1^ For instance, the guideline forming the basis for many in‐hospital resuscitation policies in the UK states “if no explicit decision has been made in advance about CPR and the express wishes of a person are unknown and cannot be ascertained, there should be an initial presumption that healthcare professionals will make all reasonable efforts to resuscitate the person in the event of cardiac or respiratory arrest.”^2^ However, a 2012 evaluation showed that for 85% of patients who were resuscitated in UK hospitals, CPR was medically futile (a majority already had a fatal underlying condition) or not in the patient's best interest.^3^ In such cases, patients often survive with a poor quality of life or the process of dying is prolonged.

The rationale behind performing a “slow code” is that it might avoid the harm of performing CPR in those cases. Clinicians may engage in this practice when they find resuscitation futile or harmful but still have to perform it as a default action because resuscitation was not discussed with the patient and next‐of‐kin before the cardiac arrest or because patients and families have unrealistic expectations and insist on CPR.^4^ Proponents of the ‘slow code’ find that intentionally delaying CPR might, in some cases, be a more compassionate alternative to aggressive and potentially futile interventions.^5^ Conversely, critics assert that ‘slow codes’ violate the fundamental principle of beneficence, in the sense of not giving the patient a true chance to survive after cardiac arrest, but moreover, that a dishonest resuscitation attempt undermines transparency and trust in the patient–physician relationship as well as in the medical profession more broadly.^6^

The debate over “slow codes” usually centers around ethical principles, and dilemmas in choosing between them, rather than on the regulations and policies that cause them to be practised in the first place. In this paper, we argue that slow codes are a symptom of ethically and legally inappropriate policies. We track the history of the development of CPR to show the discrepancy between technical progress and insufficiently implemented ethical and legal guidance in the clinical context. Once adequate policies and training of clinicians are in place, the concept of ‘slow codes’ will no longer be needed.

## 1 THE DEVELOPMENT OF CPR

While there are sporadic accounts of attempted resuscitation since antiquity,^7^ before 1960, successful resuscitation was largely limited to occasional victims of respiratory arrest.^8^ For a number of scientific and religious reasons, it was long considered impossible, even blasphemous, to attempt to reverse “death.”^9^ However, with the rapid increase of scientific discovery and the rise of secularism during the Enlightenment in the 18th century, and with good evidence that it was at this time that the doctor's duty to prolong life first emerged,^10^ the stage was finally set for progress in the area of resuscitation. Over the next 200 years, the main elements of resuscitation (artificial respiration, artificial circulation and defibrillation) were developed and eventually brought together to create the procedure of CPR.^11^

The creation of CPR formally took place on September 16, 1960, when Safar, Jude and Kouwenhoven presented their findings at the Maryland Medical Society. In this presentation, they stressed the importance of combining artificial respiration and artificial circulation, stating that the two techniques of mouth‐to‐mouth ventilation and external chest compression “cannot be considered any longer as separate units, but as parts of a whole and complete approach to resuscitation.”^12^ Although this new approach was still without a name, CPR had been born. CPR could now be used to attempt to provide an adequate circulation of oxygenated blood to keep the heart and brain of the victim alive long enough for the cardiac arrest to be reversed. However, what CPR could not do was to put the heart back into a normal rhythm. For CPR to gain widespread acceptance, there also needed to be a way of re‐establishing a normal cardiac rhythm.^13^

The main way of re‐establishing a normal cardiac rhythm is to pass an electric current through the heart to cause the complete depolarization of the myocardial fibers, in an attempt to bring about the electrical conversion of the disorganized electrical activity.^14^ Although the connection between the condition of ventricular fibrillation and electrical cardioversion was discovered in 1899, it wasn't until 1947 that Claude Beck constructed the first internal defibrillator in 1947.^15^ The ability to defibrillate the heart without having the victim's chest open, however, did not come until Paul Zoll developed external defibrillation in 1955.^16^ However, both of these defibrillators utilized alternating current (AC) and were very large and heavy. The portability issue was solved in the early 1960s when Bernard Lown developed a defibrillator that used direct current (DC) instead of AC, thus eliminating the need for a heavy transformer, allowing the defibrillator to be battery‐operated and thus travel to the patient.^17^

By the early 1960s, therefore, the medical profession was in possession of both CPR and defibrillation. With CPR now providing a means of keeping someone having cardiac arrest alive longer, and the defibrillator now being able to be rushed to the patient, there are now many more opportunities to avoid sudden cardiac death.^18^ Indeed, these two procedures have become so closely associated that the use of defibrillation is generally assumed when CPR is spoken of.

## 2 THE CHANGING USE OF CPR

CPR was initially used very selectively in a hospital setting: at the discretion of the doctor and generally on patients with acute illnesses whose cardiac arrests resulted from reversible conditions. This selective use of CPR was linked to the fact that training in its use was fairly restricted, being provided mainly for those doctors whose patients were most likely to have reversible causes of cardiac arrest (cardiologists, surgeons and anesthetists).^19^ For the most part, CPR was used only for hospital patients who had “hearts too good to die.”^20^

In 1965, the introduction to a monograph on CPR stated:

Resuscitation of the dying patient with irreparable damage to the heart, lungs, brain, or any other vital system of the body has no medical, ethical, or moral justification. The techniques described in this monograph are designed to resuscitate the victims of acute insult, whether it be from drowning, electrical shock, untoward effect of drugs, anaesthetic accident, heart block, acute myocardial infarction or surgery.^21^

Later in the same volume, two of the founders of CPR spelt out principles for its use. The first was as follows:

1. *The patient must be salvable*. Cardiopulmonary resuscitation is indicated for the patient who, at the time of cardiopulmonary arrest, is not in the terminal stage of an incurable disease. Resuscitative measures on terminal patients will, at best, return them to the dying state. The physician should concentrate on resuscitating patients who were in good health preceding the arrest, and who are likely to resume a normal existence.^22^

With the increased training of clinicians in CPR, and the development of “code teams” and “code protocols,” the use of CPR expanded rapidly to the point where it was begun promptly for all patients having cardiac arrest in the hospital, regardless of the underlying illness.^23^ One writer states that these changes were instituted “to improve the chances of a response to cardiopulmonary resuscitation, and to ensure good neurologic function in patients who did respond.”^24^ Another suggests that the “ever‐present legal threat for failure to resuscitate” was also an important factor.^25^ However, problems resulted from the use of CPR on all hospital patients who were having cardiac arrest.^26^ It was reported that “far more often than not CPR transiently restored physiologic stability but prolonged patient suffering.”^27^ In the late 1960s, reports describing the agony that many patients experienced from CPR that only prolonged their dying appeared in the medical literature.^28^ Consequently, many practitioners did not commence CPR, or performed less than a full attempt (i.e. a slow code), when they considered CPR inappropriate.^29^

In response, hospitals began to develop their own means of indicating that CPR was not to be used on a particular patient if they had a cardiac arrest. As it has been noted:

At some institutions, these decisions were concealed as purple dots on the medical record or written as cryptic initials in the patient's chart, whereas at other institutions, they were simply communicated as verbal orders passed on from shift to shift…^30^

With significant changes in the doctor–patient relationship have occurred, including a much greater emphasis on patient autonomy and shared decision‐making (see footnote 3), these practices led to controversy.^31^ There were concerns about patients not being significantly involved in decisions to withhold CPR and about the lack of documentation and transparency of these decisions.^32^ Consequently, a more formal process began to be introduced in the early 1970s.^33^ The first hospital policies on do not resuscitate (DNR) orders were reported in the medical literature in 1976^34^ and soon spread to other hospitals.^35^ These changes led to modifications of CPR policies and to a requirement that CPR be attempted on all hospital patients who were having cardiac arrest unless a DNR order was in place.^2–4,8,15^ Policies relating to DNR orders evolved over the years. By the late 1980s, many hospital policies allowed doctors to put in place a physician‐initiated DNR order for particular patients if, in their judgment, it would be futile to attempt CPR on those patients if a cardiac arrest occurred.^36^ Such policies have been maintained and have spread widely around the globe.^37^ However, CPR remains the default for all patients experiencing cardiac arrest in the hospital, regardless of their underlying illness or disease trajectory, leaving clinicians with little choice but to perform CPR if no DNR order is in place.

### 2.1 Medical practices at the end‐of‐life under current CPR policies

Death must eventually come to us all. Yet, in the words of Daniel Callahan:

The ambition of modern medicine has been to do something about that seemingly unalterable fact. It has declared war on death, on the ravages of time, and most of all on the nature that brings them about.^38^

Medicine has, indeed, had much success in this. As Callahan points out, “Life has been lengthened and death increasingly pushed into old age. People live who would once have died.” However, medicine has failed at one critical point, “that of managing the passage between life and death.”^39^ Indeed, outcomes of CPR in the hospital setting, health practitioners' views and the ‘slow code’ phenomenon clearly suggest that the current use of CPR is not conducive to good medical practices at the end of life.

The overall short‐term success rate (the return of spontaneous circulation for at least one hour) of CPR for all in‐hospital cardiac arrests is only about 40%;^40^ however, the overall long‐term success rate (survival until discharge from the hospital) of CPR for all in‐hospital cardiac arrests is particularly low, about 14%. The impressive 70% long‐term success rate that Kouwenhoven et al. initially achieved was long forgotten.^41^ Evidence also indicates that one‐third to one‐half of these survivors will have new, moderate to severe, functional or neurological impairment.^42^ However, these outcomes are overall rates for all CPR occurring in the hospital setting. CPR is in fact performed on very heterogeneous patient populations in the hospital, who have cardiac arrests for equally heterogeneous reasons. Because of this, “the potential benefits of cardiopulmonary resuscitation, and the likelihood of failure or adverse effects, are not the same for everyone.”^43^ There are several factors that are very good predictors of what the outcome of CPR will be. These include such things as the mode of the cardiac arrest, the primary rhythm of the cardiac arrest, concurrent cardiac and vascular diseases, the hospital location and the level of patient monitoring.^44^ It is clear that CPR is currently being performed on many patients who have little, or no, chance of survival, short‐term or long‐term, under the current application of CPR.^45^ It is from this situation that ‘slow codes’ flow.

Slow codes now tend to occur where there is no DNR order in place, and thus where CPR is expected to be performed, but where clinicians see attempting CPR on the patient having the cardiac arrest as clearly inappropriate and futile. This situation can cause clinicians “moral distress.”^46^ In such a situation, health practitioners may view a slow code as, “the only way of gaining some control over the situation and escaping in some way from participating in a perceived immoral act,”^47^ and as “the least bad solution.”^48^ A slow code is seen by some as an adaptive response from health practitioners to what they see as an untenable requirement of their job.^49^

## 3 SLOW CODES ARE SYMPTOMATIC OF ETHICALLY AND LEGALLY INAPPROPRIATE CPR POLICIES

After examining the changing application of CPR in the hospital setting since its development, we can see that the switch from using CPR very selectively at the discretion of the doctor, to CPR being the required default position for all patients having cardiac arrest in the hospital, was of fundamental importance. Although advance care planning could certainly be improved in most hospitals, it is clear that “slow codes” are in fact symptomatic of the more fundamental issue of having CPR as the required default position for *all* patients having cardiac arrest. On closer inspection, we find these policies to be ethically and legally inappropriate.

### 3.1 Concept of futility and CPR

The fact that a particular medical treatment is futile in a certain circumstance is widely considered to have a number of significant ethical implications.^50^ Among other things, there is generally no ethical obligation to initiate or continue such medical treatment.^51^ Indeed, some go even further than this, arguing that there is actually an ethical obligation not to provide such medical treatment and that to provide them may constitute a form of negligence.^52^ This shift in ethical obligations is considered to justify the withholding or withdrawal of medical treatments, including life‐prolonging medical treatments, and to, therefore, limit the patient's autonomous choice of medical treatment.^53^

The view of clinicians that CPR is futile and inappropriate in a certain situation is a key cause of “slow codes.”^54^ However, internationally there is no consistent understanding or consensus definition of what futile means in the case of resuscitation.^55^ The concept of futility, however, is simply one that is concerned about a formal relation, that is, about whether or not a certain means *x* will achieve a certain end *y*. Thus, it is pointless to simply say that a certain action (a means) is futile, as Truog et al. point out (1992, p. 1561), “one must always ask, ‘Futile in relation to what?’,” as an action can only be futile relative to a selected goal (an end). Some have argued that the shift of ethical obligations should only occur when CPR cannot achieve its physiological goal of re‐establishing a normal cardiac rhythm, and also the goal of postponing death, of avoiding sudden cardiac death,^56^ because all the other goals incorporate value judgments, while these two goals, it is claimed, are value‐free. It is claimed that making value judgments lies outside the proper authority of the medical profession, as such judgments rightly belong to the patient and should not be used to restrict the patient's autonomous choice of medical treatment.^57^ The underlying assumption of these arguments is that if the medical profession made value judgments, then this would undermine patient autonomy and lead to a return to paternalism.^58^ As Stuart Youngner has argued:

Physicians are in the best position to know the empirical facts about many aspects of futility. I would argue, however, that all, except for physiological futility and an absolute inability to postpone death, also involve value judgments. Physicians may be best suited to frame the choices by describing prognosis and quality of life—as well as the odds for achieving them. Physicians should not offer treatments that are physiologically futile or certain not to prolong life, and they could ethically refuse patient and family requests for such treatments. Beyond that, they run the risk of ‘giving opinions disguised as data.^59^

And similarly, the recommendations of the Hastings Center Task Force report on guidelines for the termination of life‐sustaining treatment and the care of the dying state:

In the event that the patient or surrogate requests a treatment that the responsible health care professional regards as clearly futile in achieving its physiological objective and so offering no physiological benefit to the patient, the professional has no obligation to provide it. However, the health care professional's value judgment that although a treatment will produce physiological benefit, the benefit is not sufficient to warrant the treatment, should not be used as a basis for determining a treatment to be futile.^60^

According to this position, even if the patient's illness is incurable, survival until hospital discharge is unprecedented, or the quality of life after resuscitation is poor, CPR is not entirely without benefit if it achieves its physiological goal of postponing death. If it can add even a few hours to the patient's life, it is argued that the decision should fall to the patient's autonomous choice. A one‐in‐a‐hundred chance is not zero; an extra hour of life is not anything. A few extra days of life in an intensive care unit after a cardiac arrest may be, as Lantos et al. argue, “of supreme value to a dying patient.”^61^ It is, it is argued:

…uniquely the patient's choice as to what burdens he or she will risk in gambling for improbable or transient success; this is a value‐laden decision, and thus out of the scientific realm of the physician and into the realm of the autonomous patient.^62^

These two goals, however, will not provide the value‐free sanctuary that some think they will. Indeed, no goal will. As Sulmasy (1997, p. 74) reminds us, “the setting of a goal is not empirical. It is a human choice.”^63^ The selection of *any* goal, including these two goals, as cardiopulmonary resuscitation's legitimate goal, is going to incorporate a value judgment; “goals are statements of value.”^64^

Yet, it would be a grave mistake to think that it is outside the proper authority of the medical profession to be making *any* value judgments. While some may find it attractive, a value‐free medicine is, as Cassell has pointed out, “a contradiction in terms.”^65^ Medicine is an expression of value. There are countless instances where the medical profession necessarily makes legitimate value judgments—indeed, situations where it cannot avoid doing so. Prohibiting the medical profession from making certain value judgments would lead to implausible implications for medical practice. Scientists and physicians assess the impact of probabilities and certainties, the amount and the relation of risks and benefits. Epistemic and non‐epistemic values play an important role in delineating the quality of knowledge.

The judgment of whether a particular medical treatment is going to be successful or not, for instance, will also always be one of probability, not certainty. Some degree of doubt will always be present.^66^ Yet, while there exists some small chance of success, a value judgment must be made whether this chance is *worth* pursuing or not. We recognize the authority of the medical profession to make this value judgment in a number of instances. This could apply in situations where shared decision‐making is not possible, no advance directive exists and doctors have observed progressive deterioration despite all therapeutic measures, with signs of the dying phase becoming increasingly evident. Here, empirical knowledge and expertise provide deeper insights than evidence alone. It broadens the scope of knowledge, and it is part of the professional responsibility to include this information in a value‐led assessment. Physicians should acknowledge that value judgments are being made, rather than merely ‘objective’ or ‘scientific’ medical judgments.

The judgment of whether CPR is going to be successful or not is also clearly a matter of probability. As Tomlinson and Brody write:

…it would only rarely, if ever, be the case that a physician could predict with absolute certainty that a patient would not survive cardiac arrest and resuscitation. Even for patients with conditions for which survival with CPR is unprecedented, the prediction that this next patient will also not survive is only the assertion of a high probability, not a logical certainty.^67^

If the medical profession were not permitted to make any value judgments, it would lead to the implausible scenario where clinicians could not decide when to cease a CPR attempt, even if there was no longer a reasonable chance of survival *worth* pursuing. Yet, as Tomlinson and Brody point out:

Contrary to that conclusion, however, everyone recognizes that there comes a time, short of physical exhaustion, when hope for success is no longer reasonable and when the physician may stop…This justifiable decision to stop is not based on any claim of “absolute” certainty, as Youngner would have it, because there is no such thing. It can only be based on a shared, value‐laden understanding of what counts as a reasonable chance worth pursing…^68^

Sooner or later, death is the inevitable outcome for us all; the “intrinsic mortality of the human condition”^69^ ought not to be forgotten in all of this. Because of its necessary place in human life, death *per se* should not be considered by medicine as the “enemy.” As Gebhard et al. write:

It is death at the wrong time (too early in life), for the wrong reasons (medically avoidable or treatable at a reasonable cost), and coming to the patient in the wrong way (full of relievable pain and suffering and excessively prolonged) that are the appropriate enemies.^70^

Indeed, Leon Kass has gone as far as to argue that the prolongation of life, or the prevention of death, is “a false goal of medicine.”^71^ Kass has argued forcefully against redefining the primary goal of medicine as a relentless prolongation of life, stating:

If medicine takes aim at death prevention, rather than at health and relief of suffering, if it regards every death as premature, as a failure to today's medicine – but avoidable by tomorrow's—then it is tacitly asserting that its true goal is bodily immortality…Physicians should try to keep their eyes on the main business, restoring and correcting what can be corrected and restored, always acknowledging that death will and must come, that health is a mortal good, and as embodied beings we are fragile beings that must stop sooner or later, medicine or no medicine.^72^

The struggle against death needs to be cautiously balanced against the realization that the role of medicine is not to prevent death, to prolong life, at all costs, “but to help people live as healthy lives as possible within a finite life span.”^73^ Because of this, we partially agree with Callahan that respect for individual life does not demand an unlimited effort to use every technological possibility to prolong that life and that we should, in fact, be extremely reluctant to use life‐prolonging technology in the care of critically ill patients, particularly patients who are terminally ill or irreversibly declining, unless two conditions are met.^74^ First, there is a good probability that the technology will significantly modify the course of the underlying illness. Second, the long‐term outcome for the patient as a person promises to be a good one. Callahan calls the combination of these two conditions the “benefit presumption test”; “meaning to suggest that to use a technology there should be a positive presumption that the patient will benefit.”^75^ As Callahan writes:

“The goal here is to let the patient's condition and the long‐term prognosis (not the mere forestalling of death) determine whether to make use of those technologies that can artificially sustain the body. The test is what a technology will do for the overall life and welfare of a patient, not what it will do to forestall a coming death or to sustain organ systems. A cardiopulmonary resuscitation (CPR) in the case of an advanced terminally ill cancer patient…provide examples of cases where an application of the “benefit presumption test” would be appropriate …The point is to retain respect for the value of individual human life, the dignity of the life, while not becoming slaves to our technology.^76^

Although physicians do not have the legitimacy to impose their individual concepts of a good life on patients, they do have a professional duty and responsibility to distinguish between life and the prolongation of the dying process.

Ethically, CPR should be removed as the required default position for all patients regardless of their individual situation, and hospital policies should be modified to allow clinicians to consider whether CPR is appropriate at the time of arrest if there is no patient‐initiated DNR order in place. The prolongation of life at all costs is not an ethically appropriate goal of medicine, as it has already been recognized in the context of CPR with the development of DNR orders. Although improved end‐of‐life communication and advance care planning with patients will likely help reduce CPR being provided in inappropriate situations, it is unrealistic to think that advance care planning and a DNR order will be in place for all in‐hospital patients at any time. Patients are not obliged to do an advance directive, and the extent to which physicians draw up treatment plans for possible life‐threatening situations is at their discretion.

Before any medical intervention is provided to a patient, a judgment needs to be made whether the intervention is medically indicated, that is, whether there is a valid reason for using the intervention; this should be no different in the context of CPR. Determining an intervention is medically indicated is independent of patient consent and is the clinician's responsibility as the expert. Medical indication is a normative concept, requiring clinicians to balance scientific facts with individual needs and goals. As such, it must be a context‐sensitive determination made on an individual basis. Indeed, this may occasionally lead to a decision to provide CPR in circumstances that do not meet Callahan's two conditions above, for example, a terminally ill cancer patient who wishes to survive another month to witness their daughter's wedding.

Deciding whether CPR is medically indicated and in the patient's best interests in emergency situations will often be very challenging, and some may worry about clinicians' ability to accurately determine this. There is simply no escaping, however, the uncertainty that exists in these situations. The decision whether or not to initiate CPR is a life or death decision, and if there is genuine uncertainty, then CPR should be initiated promptly in the first instance. Physicians have an ethical and legal obligation to put the interests of their patients first. However, requiring CPR in circumstances where all involved agree that it is not in the patient's best interests is ethically inappropriate. As the slow‐code phenomenon demonstrates, forcing clinicians to provide CPR when they believe it is inappropriate only leads to half‐measures to manage the moral distress they experience. In situations where advance care planning has not be able to happen, the decision of whether CPR should be performed or not needs to be an individual context‐sensitive determination by clinicians at the time of arrest.

### 3.2 Importance of the patient's best interests

An important ethical and legal right of individuals is to have their bodily integrity respected. One of the consequences of this is that the provision of medical treatment without consent or some other legal justification can incur various forms of liability.^77^ A patient having cardiac arrest will clearly not have the level of competence required to consent to CPR, nor would consent have always been given in advance by the patient. For the provision of CPR to be lawful, therefore, there needs to be some kind of legal justification for providing it without consent.

The standard legal grounds for providing medical treatment without consent in common law systems generally depend upon a judgment that the provision of treatment is in the patient's best interests.^78^ Thus, in the absence of a patient's valid anticipatory refusal of consent (e.g. a patient‐initiated DNR order), the provision of CPR is lawful whenever there are reasonable grounds for believing that it is in the patient's best interests. Although clinicians will be given considerable leeway in making this assessment, often in difficult situations, the issue of whether the treatment is in the patient's best interests is key.^79^

It is therefore concerning that many current CPR policies do not allow clinicians to consider whether CPR is appropriate at the time of arrest. This approach can lead to CPR being used, “not for the good that it will do, but that nothing may be left undone on the margin of the impossible.”^80^ This approach, however, is legally inappropriate. Performing CPR without consent in circumstances where it cannot be reasonably regarded as in the patient's best interests is unlawful in most common law jurisdictions.^81^ The rapid initiation of CPR, however, is important in determining a successful outcome for a victim of a cardiac arrest.^82^ In situations where clinicians are not sure whether or not CPR is in the best interests of the patient, starting CPR at least in the first instance is sensible and supported by the law.^83^ However, there is no justification for proceeding with CPR when a DNR order has not been made, but all involved agree that further treatment is not in the patient's best interests.^84^

In a joint statement on these issues in 2007 by the British Medical Association, Resuscitation Council (UK) and Royal College of Nursing, it is noted that

There will be some patients for whom attempting CPR is clearly inappropriate; for example, a patient in the final stages of a terminal illness where death is imminent and unavoidable and CPR would not be successful, but for whom no formal DNAR decision has been made. In such circumstances, healthcare workers who make a considered decision not to commence CPR should be supported by their senior colleagues and employers.

Under hospital policies in many countries, however, clinicians are currently not given the opportunity to make a “considered decision.” This situation leads to “slow codes.”^85^ Slow codes have been decried as unethical in recent policy documents such as the 2021 ERC guidelines (“Clinicians should not partake in ‘slow codes’, p. 410”).^86^ However, further steps are required to address the continued practice of “slow codes.”

## 4 PERSPECTIVES ABOUT A FUTURE WITH CONSENTED GOALS OF CARE INSTEAD OF SLOW CODES

We find that hospital policies should be modified to allow clinicians to consider whether CPR is appropriate at the time of arrest,^87^ and clinical training should include a stronger emphasis on early recognition of patients for whom CPR is not in their best interests, so as to empower clinicians to act quickly at the “margin of (im)possibility.” However, one lesson learned during the pandemic is that “ethics cannot simply be switched on during an emergency.”^88^ We need a translation of ethical guidance into policies and context‐sensitive protocols.^89^

First, the discrepancy between recent policy statements about “slow codes” being unethical on the one hand, and the ongoing practice in hospitals on the other, may stem from a lack of communication and discussion about these policies. Research in the Netherlands, for instance, showed limited understanding of CPR policies, with almost half of surveyed clinicians incorrectly thinking that a patient‐initiated DNR statement is more of a request rather than a strict obligation for clinicians to follow.^90^ Given this general lack of understanding of resuscitation policies, training and ongoing education for professionals is needed about ethical and legal regulations related to the decision to start or stop CPR. Of note is that ethical guidance should be in concordance with a country's laws because policies that do not have CPR as a default position and that allow room for physicians' value considerations if CPR is clearly not in patients' best interest are useless if they are not legally supported; clinicians worrying over litigation might then still perform ‘slow codes’.

Second, not only guidance for clinicians but also engagement with public and patient representatives is needed for a debate on when CPR is in the best interest of the patient or, alternatively, does not harm the patient disproportionately. Empirical data suggest that there is still an ongoing misconception, a lack of knowledge and prognostic awareness about CPR in patients, sometimes fostered by television.^91^ Given that already in 1994, Murphy et al. showed that patients' preferences are influenced by the information about CPR outcomes, and the communication about representative evidence is important: only half of the about forty per cent who opted for CPR in case of acute illness maintained their position when they received transparent prognostic information.^92^ As far as we know, there is no published research about public perspectives on slow codes. Public discussions should also address the topic of death in a more fundamental way. Sociologist Stefan Timmermans has argued that CPR is part of a society that medicalises death and corrupts the dying experience.^93^ He writes: “in its pure form, sudden death is very close to our contemporary conception of a good death, while in its current form it's a death to be avoided.” While in‐hospital resuscitation might de‐socialise the dying process and harm the dignity of the person receiving CPR, confronting our death anxiety creates the opportunity for finding meaning. This confronting implies that discussions about death are needed so that people do not have unrealistic expectations and that they can engage in advance planning of their care at the end of life and their dying process.

Third, further study of and guidance on advance care planning is needed. Engaging in advanced care planning would help protect patients and families as well as clinicians^94^ and at least partly remove the felt need for “slow codes.” Since 2021, the European resuscitation guidelines have highlighted the importance of advanced care planning.^95^ To implement this recommendation, it has been suggested that there is a need to reframe advanced care planning “as part of the continuum of care planning across the life course” with patients' quality of life as the “fundamental cornerstone.”^96^ Furthermore, it has also been proposed that DNR orders should be renamed to resuscitation or treatment escalation plans for in‐hospital patients.^97^ CPR policies should encourage clinicians and patients to engage in advanced care planning as a model of shared decision‐making and should be supported by in hospital goals‐of‐care programs and interdisciplinary team‐based approaches for complex medical and ethical decisions, including information, education and training in ethics and advanced care planning, just as regular as CPR training is enacted. With these proposed changes to CPR policy and practice, we hope that ‘slow codes’ will become a thing of the past.

## Footnotes

1 Dignam, C., Brown, M., & Thompson, C. H. (2021). Moving from “do not resuscitate” orders to standardized resuscitation plans and shared‐decision making in hospital inpatients. *Gerontology and Geriatric Medicine*, *7*, 23337214211003431.

2 British Medical Association, the Resuscitation Council (UK), & the Royal College of Nursing. (2016). *Decisions relating to cardiopulmonary resuscitation* (3rd ed., 1st rev., p. 17).

3 Perkins, G. D., Temple, R. M., & George, R. (2012). Time to intervene: Lessons from the NCEPOD report. *Resuscitation*, *83*(11), 1305–1306.

4 DePalma, J. A., Ozanich, E., Miller, S., & Yancich, L. M. (1999). “Slow” code: Perspectives of a physician and critical care nurse. *Critical Care Nursing Quarterly*, *22*(3), 89–99; Piscitello, G. M., Kapania, E. M., Kanelidis, A., Siegler, M., & Parker, W. F. (2021). The use of slow codes and medically futile codes in practice. *Journal of Pain and Symptom Management*, *62*(2), 326–335 e325.

5 Lantos, J. D., & Meadow, W. L. (2011). Should the “slow code” be resuscitated? *The American Journal of Bioethics*, *11*(11), 8–12.

6 Weinacker, A. (2011). The “slow code” should be a “no code”. *The American Journal of Bioethics*, *11*(11), 27–29.

7 Ardagh, M. (2004). A brief history of resuscitation. *New Zealand Medical Journal*, *117*, http://www.nzma.org.nz/journal/117-1193/868/; New Zealand Resuscitation Council Inc. (2002). *Basic resuscitation: For health professionals*. New Zealand Resuscitation Council Inc; Eisenberg, M. S. (1996). The Quest to Reverse Sudden Death: A History of Cardiopulmonary Resuscitation. In: N. A. Paradis, H. R. Halperin, & R. M. Nowak (Eds.), Cardiac Arrest: The Science and Practice of Resuscitation Medicine (pp. 1–27). Williams & Wilkins; Paraskos, J. A. (1992). Biblical accounts of resuscitation. *The Journal of the History of Medicine and Allied Sciences*, *47*, 310–321; Hermreck, A.S. (1988). The history of cardiopulmonary resuscitation. *American Journal of Surgery*, *156*, 430–436.

8 Emergency Cardiac Care Committee and Subcommittees of the American Heart Association. (1992). Guidelines for cardiopulmonary resuscitation and emergency cardiac care. *JAMA*, *268*, 2135–2302.

9 McLennan, S. (2008). The development of CPR. *NZMJ*, *121*(1284), 71–77.

10 Ibid; Amundsen, D. W. (1978). The physician's obligation to prolong life: A medical duty without classical roots. *Hastings Centre Report*, *8*, 23–30.

11 ; Eisenberg, op. cit. note 7; Hermreck, op. cit. note 7.

12 Eisenberg, op. cit. note 7, p. 13.

13 Eisenberg, op. cit. note 7.

14 New Zealand Resuscitation Council Inc, op. cit. note 7; Jude, J. R., & Elam, J. O. (1965). *Fundamentals of cardiopulmonary resuscitation*. Davis Company.

15 Beck, C. S., Pritchard, W. H., & Feil, H. S. (1947). Ventricular fibrillation of long duration abolished by electrical shock. *JAMA*, *135*, 985–986.

16 Zoll, P. M., Linenthal, A. J., Gibson, W., Paul, M. H., & Norman, L. R. (1956). Termination of ventricular fibrillation in man by externally applied electric countershock. *New England Journal of Medicine*, *254*, 727–732.

17 Eisenberg, op. cit. note 1; Lown, B., Amarasingham, R., & Neuman, J. (1962). New method for terminating cardiac arrhythmias: Use of synchronised capacitor discharge. *JAMA*, *182*, 548*–*555.

18 Eisenberg, op. cit. note 7.

19 New Zealand Resuscitation Council Inc, op. cit. note 7; Faber‐Langendoen, K. (1991). Resuscitation of patients with metastatic cancer: Is transient benefit still futile? *Archives of Internal Medicine*, *151*, 235–239; Blackhall, L. J. (1987). Must we always use CPR? *New England Journal of Medicine*, *317*, 1281–1285.

20 Eisenberg, M. S., & Mengert, T. J. (2001). Cardiac resuscitation. *New England Journal of Medicine*, *344*, 1304–1313, p. 1311.

21 Talbott, J. H. (1965). Introduction. In: J. R. Jude & J. O. Elam (Eds.), *Fundamentals of cardiopulmonary resuscitation* (pp. 1–3). Davis Company, p. 3.

22 Jude & Elam, op. cit. note 14.

23 Blackhall, op. cit. note 19.

24 Ibid, p. 1282.

25 Hermreck, op. cit. note 7, p. 435.

26 Burns, J. P., Edwards, J., Johnson, J., Cassem, N. H., & Truog, R. D. (2003) Do‐not‐resuscitate order after 25 years. *Critical Care Medicine*, *31*, 1543–1550; Blackhall, op. cit. note 19; Hermreck, op. cit. note 7.

27 Burns, J. P., et al., op. cit. note 26, p. 1543.

28 E.g., Symmers, W. S. (1968). Not allowed to die. *British Medical Journal*, *1*, 442.

29 Burns, J. P., et al., op. cit. note 26.

30 Burns, J. P., et al., op. cit. note 26, p. 1543.

31 Faber‐Langendoen, op. cit. note 19.

32 Burns, J. P., et al., op. cit. note 26, p. 1543.

33 National Conference on Cardiopulmonary Resuscitation and Emergency Cardiac Care. (1974). Standards and guidelines for cardiopulmonary resuscitation and emergency cardiac care: Medicolegal considerations and recommendations. *JAMA*, *227*, 837–868.

34 Clinical Care Committee of the Massachusetts General Hospital. (1976). Optimum care for hopelessly ill patients: A report of the Clinical Care Committee of the Massachusetts General Hospital. *New England Journal of Medicine*, *295*, 362–364; Rabkin, M. T., Gillerman, G., & Rice, N. R. (1976). Orders not to resuscitate. *New England Journal of Medicine*, *295*, 362–364; Fried, C. (1976). Terminating life support: Out of the closet! *New England Journal of Medicine*, *295*, 390–391.

35 Burns, J. P., et al., op. cit. note 26.

36 Swig, L., Cooke, M., Osmond, D., Luce, J. A., Brody, R. V., Bird, C., & Luce, J.M. (1996). Physician Responses to a Hospital Policy Allowing Them to Not Offer Cardiopulmonary Resuscitation. *Journal of the American Geriatrics Society*, *44*, 1215–1219.

37 Ditillo, B. A. (2002). Should there be a choice for cardiopulmonary resuscitation when death is expected? Revisiting an old idea whose time is yet to come. *Journal of Palliative Medicine*, *5*, 107–116; Swig, L., et al., op. cit., note. 36; Tomlinson, T., & Czlonka, D. (1995). Futility and hospital policy. *Hastings Center Report*, *25*, 28–35; Waisel, D. B., & Truog, R. D. (1995). The cardiopulmonary resuscitation‐not‐indicated order: Futility revisited. *Annals of Internal Medicine*, *122*, 304–308.

38 Callahan, D. (1993). *The troubled dream of life: Living with mortality* (p. 57). Simon and Schuster.

39 Ibid:58.

40 Peberdy, M. A., Kaye, W., Ornato, J. P., Larkin, G. L., Nadkerni, V., Mancini, M. E., Berg, R. A., Nichol, G., & Lane‐Trultt, T. (2003). Cardiopulmonary resuscitation of adults in the hospital: A report of 14720 cardiac arrests from the National Registry of Cardiopulmonary Resuscitation. *Resuscitation*, *58*, 297–308; Cooper, S., & Evans, C. (2003). Resuscitation Predictor Scoring Scale for inhospital cardiac arrests. Emergency Medical Journal, 20, 6‐9.

41 Conroy, S. P., Luxton, T., Dingwall, R., Harwood, R. H., & Gladman, J. R. F. (2006). Cardiopulmonary resuscitation in continuing care settings: time for a rethink? *British Medical Journal*, 332, 479–482; Peberdy, M. A., et al., op. cit. note. 40; Cooper & Evans, op. cit. note 40; New Zealand Resuscitation Council Inc, op. cit. note 7; Ebell, M. H., Becker, L. A., Barry, H. C., & Hagen, M. (1998). Survival after in‐hospital cardiopulmonary resuscitation: A meta‐analysis. *Journal of General Internal Medicine*, *13*, 805–816; Diem, S. J., Lantos, J. D., & Tulsky, J. A. (1996). Cardiopulmonary resuscitation on television: Miracles and misinformation. *New England Journal of Medicine*, *334*, 1578–1582; Ballew, K. A., & Philbrick, J. T. (1995). Causes of variation in reported in‐hospital CPR survival: A critical review. *Resuscitation*, *30*, 203–215; Valentin, A., Karnik, R., Donath, P., Winkler, W. B., & Slany, J. (1995). Outcome of cardiopulmonary resuscitation in hospitalized patients. *Resuscitation*, *30*, 217–221; Murphy, D. J., Murry, A. M., Robinson, B. E., & Campion, E. W. (1995). Outcomes of cardiopulmonary resuscitation in the elderly. *Annals of Internal Medicine*, *122*, 368–374; Kouwenhoven, W. B., Jude, J. R., & Knickerbocker, G. G. (1960). Closed‐chest cardiac massage. *JAMA*, *173*, 1064–1067.

42 Peberdy, M. A., et al., op. cit. note. 40; De Vos, R. R. N., Koster, R., & de Haan, R. (1999). Quality of survival after cardiopulmonary resuscitation. *Archives of Internal Medicine*, *159*, 249–254; FitzGerald, J. D, Califf, R. M., Phillips, R. S., Desbiens, N. A., Liu, H., Lynn, J., Wu, A. W., Connors, A. F., & Oye, R. K. (1997). Functional status among survivors of in‐hospital cardiopulmonary resuscitation. *Archives of Internal Medicine*, *157*, 72–76; Valentin, A., et al., note 41; Longstreth, W. T., Cobb, L. A., & Copass, M. K. (1983). Neurologic recovery after out‐of‐hospital cardiac arrest. *Annals of Internal Medicine*, *5*, 588–592.

43 Conroy, S. P., et al., op. cit. note 41, p. 479.

44 Ardagh, M. (1996). Resuscitation from out‐of‐hospital cardiac arrest: past, present and future. *New Zealand Medical Journal*, *109*, 153–154.

45 Allen, M. B., Bernacki, R. E., Gewertz, B. L., Cooper, Z., Abrams, J. L., Peetz, A. B., Bader, A. M., & Sadovnikoff, N. (2021). Beyond the do‐not‐resuscitate order: An expanded approach to decision‐making regarding cardiopulmonary resuscitation in older surgical patients. *Anesthesiology*, *135*(5), 781–787.

46 Corley, M. C. (1995). Moral distress of critical care nurses. *American Journal of Critical Care*, *4*, 280–285.

47 Ditillo, op. cit. note 37.

48 Lantos & Meadow, op. cit. note 5.

49 Muller, J. H. (1992). Shades of blue: The negotiation of limited codes by medical residents. *Social Science and Medicine*, *34*, 885–898.

50 Lantos, J. D., Singer, P. A., Walker, R. M., Gramelspecher, G. P., Shapiro, G. R., Sanchez‐Gonzalez, M. A., Stocking, C. B., Miles, S. H., & Siegler, M. (1989). The illusion of futility in clinical practice. *American Journal of Medicine*, *87*, 81–84; Ramsey, P. (2002). *The patient as person: explorations in medical ethics* (2nd ed.) Yale University Press; Beauchamp, T., & Childress, J. (2001). *Principles of biomedical ethics* (5th ed.). Oxford University Press; Sulmasy, D. (1997). Futility and the varieties of medical judgment. *Theoretical Medicine*, *18*, 63–78; Hilberman, M., Kutner, J., Parsons, D., & Murphy, D. J. (1997). Marginally effective medical are: Ethical analysis of issues in cardiopulmonary resuscitation (CPR). *Journal of Medical Ethics*, *23*, 361–367; Schneiderman, L. J., Jecker, N. S., & Jonsen, A. R. (1996). Medical futility: Response to critiques. *Annals of Internal Medicine*, *125*, 669–674; Luce, J. M. (1995). Physicians do not have a responsibility to provide futile or unreasonable care if a patient or family insists. *Critical Care Medicine*, *23*, 760–766; Alpers, A., & Lo, B. (1995). When is CPR futile? *JAMA*, *273*,156–158; Jecker, N. S., & Schneiderman, L. J. (1993). Medical futility: The duty not to treat. *Cambridge Quarterly of Healthcare Ethics*, *2*, 151–159; Faber‐Langendoen, op. cit. note 19; Tomlinson, T., & Brody, H. (1990). Futility and the ethics of resuscitation. *JAMA*, *264*, 1276–1280; Hackler, C., & Hiller, C. (1990). Family consent to orders not to resuscitate: Reconsidering hospital policy. *JAMA*, *264*, 1281–1283; Schneiderman, L. J., Jecker, N. S., & Jonsen, A. R. (1990). Medical futility: Its meaning and ethical implications. *Annals of Internal Medicine*, *112*, 949–954; Murphy, D. J. (1988). Do‐not‐resuscitate orders: Time for reappraisal in long‐term‐care institutions. *JAMA*, *260*, 2098–2101; Blackhall, op. cit. note 19; Brett, A. S., & McCullough, L. B. (1986). When patients request specific interventions: defining the limits of the physician's obligation. *New England Journal of Medicine*, *315*, 1347–1351; McCormick, R. A. (1974). To save or to let die: The dilemma of modern medicine. *JAMA*, *229*, 172–176.

51 Ibid.

52 Ramsey, op. cit. note 50; Jecker & Schneiderman, op. cit. note 50.

53 Beauchamp & Childress, op. cit. note 50, Lantos, J. D., op. cit. note 50; Blackhall, op. cit. note 19; Tomlinson & Brody, op. cit. note 50; Murphy, op. cit. note 50; Ramsey, op. cit. note 50; Sulmasy, op. cit. note 50; Hilberman, M., et al., op. cit. note 50; Schneiderman, L. J., et al., op. cit. note 50; Luce, op. cit. note 50; Alpers & Lo, op. cit. note 50; Jecker & Schneiderman, op. cit. note 50; Faber‐Langendoen, op. cite. note. 27; Hackler & Hiller, op. cit. note 50; Schneiderman, L. J., et al., op. cit. note 50; Brett & McCullough, op. cit. note 50; McCormick, op. cit. note 50.

54 Piscitello, G. M., et al., op. cit. note 4.

55 Beck, K., Vincent, A., Cam, H., Becker, C., Gross, S., Loretz, N., Müller, J., Amacher, S. A., Bohren, C., Sutter, R., Bassetti, S., Hunziker, S. (2022). Medical futility regarding cardiopulmonary resuscitation in in‐hospital cardiac arrests of adult patients: A systematic review and Meta‐analysis. *Resuscitation*, *172*, 181–193. https://doi.org/10.1016/j.resuscitation.2021.11.041

56 Waisel & Truog, op. cit. note 37; Youngner, S. J. (1988). Who defines futility? *JAMA*, *260*, 2094–2095; Hastings Center. (1987). *Guidelines on the termination of life‐sustaining treatment and the care of the dying*. Indiana University Press.

57 Ibid.

58 Tomlinson & Brody, op. cit. note 50.

59 Youngner, op. cite. note 56, pp. 2094–2095.

60 Hastings Center, op. cit. note 56, p. 32.

61 Lantos, J. D., op. cit. note 50, p. 83.

62 Faber‐Langendoen, op. cit. note 19, p. 237.

63 Sulmasy, op. cit. note 50.

64 Cassell, E. J. (2004). *The nature of suffering and the goals of medicine* (p. 173). Oxford University Press.

65 Cassell, op. cit. note 64, p. 173.

66 Sulmasy, op. cit. note 50.

67 Tomlinson & Brody, op. cit. note 50, p. 1276.

68 Ibid:1278.

69 Gebhard, A., Blasszauer, B., Boyd, K., Callahan, D. (1996a). The goals of medicine: Looking forward. *The Hastings Center Report*, *26*, S23–S25, p. S24.

70 Gebhard, A., Blasszauer, B., Boyd, K., Callahan, D. (1996b). The goals of medicine: Specifying the goals of medicine. *The Hastings Center Report*, *26*, S9–S14, p. S14.

71 Kass, L. R. (1985). *Towards a more natural science: Biology and human affairs*. Free Press.

72 Kass, L.R. (1980). Ethical dilemmas in the care of the ill. II: What is the patient's good? *JAMA*, *244*, 1947.

73 Gebhard, A., et al., op. cit. note 70, p. S24.

74 Callahan, D. (1990). *What kind of life: The limits of medical progress*. Simon and Schuster.

75 Ibid:248.

76 Ibid.

77 McLennan, S. (2012). CPR policies and the patient's best interests. *Journal of Resuscitation*, *83*, 168–170.

78 Ibid; Kopelman, L. M. (2007). The best interests standard for incompetent or incapacitated persons of all ages. *Journal of Law, Medicine & Ethics*, *35*, 187–196.

79 McLennan, S., Paterson, R., Skegg, P. D. G., & Aickin, R. (2011). The use of CPR in New Zealand: Is it always lawful? *New Zealand Medical Journal*, 124, 106–112.

80 Eliot, T. S. (1962). *The family reunion*. Faber and Faber.

81 McLennan, op. cit. note 77.

82 Rea, T. D., Bobrow, B. J., Edelson, D. P., Berg, R. A., Sayre, M. R., Berg, M. D., Chameides, L., O'Connor, R. E., & Swor, R. A. (2020). Part 4: CPR overview: 2010 American heart association guidelines for cardiopulmonary resuscitation and emergency cardiovascular care. *Circulation*, 122, S676–S684.

83 McLennan, op. cit. note 77.

84 Ibid.

85 Ganz, F. D., Sharfi, R., Kaufman, N., & Einav, S. (2019). Perceptions of slow codes by nurses working on internal medicine wards. *Nursing Ethics*, *26*, 1734–1743; Einav, S., Avidan, A., Brezis, M., & Rubinow, A. (2006). Attitudes of medical practitioners towards “Do Not Resuscitate” orders. *Medicine and Law*, *25*, 219–228; Vandeplassche, S., & van de Voorde, P. (2018). Retrospective population‐based study of emergency medical services‐attended out‐of‐hospital cardiac arrests in children in Belgium. *European Journal of Emergency Medicine*, *25*, 400–403.

86 Mentzelopoulos, S. D., Couper, K., Van de Voorde, P., Druwé, P., Blom, M., Perkins, G. D., Lulic, I., Djakow, J., Raffay, V., Lilja, G., & Bossaert, L. (2021). European Resuscitation Council Guidelines 2021: Ethics of resuscitation and end of life decisions. *Resuscitation*, *161*, 408–432.

87 McLennan, op. cit. note 77.

88 Emanuel, E. J., Upshur, R. E. G., & Smith, M. J. (2022). What Covid Has taught the world about ethics. *New England Journal of Medicine*, *387*(17), 1542–1545.

89 Knochel, K., Schmolk, E. M., Meier, L., & Buyx, A. (2024). Translating theories of justice into a practice model for triage of scarce intensive care resources during a pandemic. *Bioethics*, *38*(3), 223–232.

90 van Delden, J. J. M., van der Heide, A., van de Vathorst, S., Weyers, H., & van Tol, D. G. (2011). Knowledge and views of public and professionals on medical decision‐making and treatment around the end of life The KOPPEL study [Original Dutch: Kennis en opvattingen van publiek en professionals over medische besluitvorming en behandeling rond het einde van het leven Het KOPPEL‐onderzoek].

91 Heyland, D. K., Frank, C., Groll, D., Pichora, D., Dodek, P., Rocker, G., & Gafni, A. (2006). Understanding cardiopulmonary resuscitation decision making: perspectives of seriously ill hospitalized patients and family members. *Chest*, *130*(2), 419–428. https://doi.org/10.1378/chest.130.2.419; Bandolin, N. S., Huang, W., Beckett, L., & Wintemute, G. (2020) Perspectives of emergency department attendees on outcomes of resuscitation efforts: origins and impact on cardiopulmonary resuscitation preference. *Emergency Medicine Journal*, *37*, 611–616; Diem, S. J. et al., op. cit. 41.

92 Murphy, D. J., Burrows, D., Santilli, S., Kemp, A. W., Tenner, S., Kreling, B., & Teno, J. (1994). The influence of the probability of survival on patients' preferences regarding cardiopulmonary resuscitation. *New England Journal of Medicine*, *330*(8), 545–549.

93 Timmermans, S. (1999). *Sudden death and the myth of CPR* (p. 28). Temple University Press.

94 McMahan, R. D., Hickman, S. E., & Sudore, R. L. (2024). What Clinicians and researchers should know about the evolving field of advance care planning: A narrative review. *Journal of General Internal Medicine*, *39*(4), 652–660.

95 Mentzelopoulos, S.D. et al., op. cit. note 86.

96 Hickman, S. E., Lum, H. D., Walling, A. M., Savoy, A., & Sudore, R. L. (2023). The care planning umbrella: The evolution of advance care planning. *Journal of the American Geriatrics Society*, *71*(7), 2350–2356.

97 Dignam, C., et al., op. cit. note 1; Warner, B. E., Lound, A., Grailey, K., Vindrola‐Padros, C., Wells, M., & Brett, S. J. (2023). Perspectives of healthcare professionals and older patients on shared decision‐making for treatment escalation planning in the acute hospital setting: A systematic review and qualitative thematic synthesis. *EClinicalMedicine*, *62*, 102144. https://doi.org/10.1016/j.eclinm.2023.102144

